# SIVIC: Open-Source, Standards-Based Software for DICOM MR Spectroscopy Workflows

**DOI:** 10.1155/2013/169526

**Published:** 2013-07-18

**Authors:** Jason C. Crane, Marram P. Olson, Sarah J. Nelson

**Affiliations:** Surbeck Laboratory for Advanced Imaging, Department of Radiology and Biomedical Imaging, University of California, San Francisco, CA 94158-2330, USA

## Abstract

Quantitative analysis of magnetic resonance spectroscopic imaging (MRSI) data provides maps of metabolic parameters that show promise for improving medical diagnosis and therapeutic monitoring. While anatomical images are routinely reconstructed on the scanner, formatted using the DICOM standard, and interpreted using PACS workstations, this is not the case for MRSI data. The evaluation of MRSI data is made more complex because files are typically encoded with vendor-specific file formats and there is a lack of standardized tools for reconstruction, processing, and visualization. SIVIC is a flexible open-source software framework and application suite that enables a complete scanner-to-PACS workflow for evaluation and interpretation of MRSI data. It supports conversion of vendor-specific formats into the DICOM MR spectroscopy (MRS) standard, provides modular and extensible reconstruction and analysis pipelines, and provides tools to support the unique visualization requirements associated with such data. Workflows are presented which demonstrate the routine use of SIVIC to support the acquisition, analysis, and delivery to PACS of clinical ^1^H MRSI datasets at UCSF.

## 1. Introduction

MR spectroscopic imaging (MRSI) is a powerful imaging technique that provides spatially resolved metabolic information. It has been used together with anatomical and functional imaging to improve diagnostic specificity in multiple diseases, and it shows promise for improving treatment planning and the ability to monitor therapeutic response [1–11].

Despite great interest in this technology from the research and clinical communities, the adoption of advanced MRSI methods has been relatively slow, with a relatively limited number of studies having applied such techniques in clinical trials of new therapies. A major limitation in integrating MRSI into these studies has been the lack of commercially available methods for visualization and interpretation of the data. For conventional 3D imaging, the use of the DICOM [12] standard has resulted in a great deal of interoperability between software packages, imaging archives, and data. However, despite the existence of a DICOM standard for encoding MRSI data [13], current datasets are still created with vendor-specific proprietary formats. This results in a low degree of interoperability between imaging devices, picture archiving and communication systems (PACS), and software packages for analyzing the data. This situation is particularly problematic for multicenter collaborations, which require complicated workflows and file format conversions to evaluate data from multiple vendors. As a result, information about variations in metabolic parameters is typically delivered to PACS in the form of static DICOM secondary capture images, which hinders its integration with other types of multimodal imaging data [3]. This hinders the development and validation of postprocessing methodologies as well as the integration of MRSI data into routine radiological workflows. 

The open-source software package known as SIVIC (Spectroscopic Imaging, VIsualization, and Computing) [14, 15] was developed at UCSF to address the limitations of existing strategies for analyzing MRSI data. In the following, there is firstly an overview of MRSI data, followed by a description of the SIVIC software package. Two workflows that have been implemented at UCSF in order to streamline the routine use of MRSI in research and clinical studies are presented as examples of the applications of SIVIC. This is followed by a description of an approach for generalizing MRSI data analysis pipelines.

## 2. Features of MRSI Data

**Figure 1:**
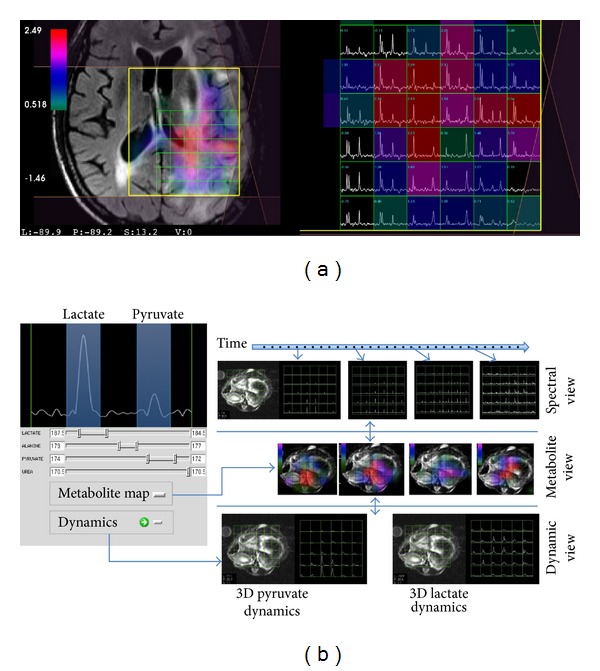
Multidimensional MRSI data visualization. (a) 4D brain MRSI data in SIVIC. Spectra from individual voxels are shown on the right. The left panel shows the spatial localization of each MRSI voxel on a reference anatomical image. The color overlay is a 3D metabolite map derived from spectral quantification of individual peaks. (b) 5D dynamic MRSI data. Metabolite peaks are derived from each point in a time series of 4D MRSI volumes. 3D dynamics of individual metabolites are represented by time curves in the bottom row for two different metabolites. The example at the bottom is from hyperpolarized  ^13^C MRSI of a rat.

Working with MRSI data has unique requirements compared with anatomical and functional images. In a volumetric sense, MRSI data is at least 4-dimensions, comprising 3 spatial and at least one spectral dimension. Dynamic and multichannel MRSI acquisitions result in data with 5 or more dimensions. Reconstruction, postprocessing, and quantification of such data require specialized algorithms for generating and evaluating spectral data. Once reconstructed, the MRSI data are typically visualized by displaying a frequency spectrum at each spatial location (Figure 1(a)). Dynamic MRSI requires analysis of MRSI data at multiple time points and is conveniently represented as frequency specific plots reflecting the dynamic behavior of individual metabolites (Figure 1(b)). This means that specialized tools are required to represent the data and correlate it with other types of images. 

MRSI data are often encoded in vendor specific formats or private DICOM SOP classes. This introduces a major obstacle in managing the data and developing software that will work with data acquired on scanners from multiple vendors. In contrast, anatomical images are typically encoded as standard DICOM MR Image Storage SOP instances. This enables existing DICOM infrastructures to be used for data transmission between devices, storage of images in PACS, and visualization with standardized image viewing applications. MRSI data, on the other hand, require special workflow protocols that are separate from the standard workflows. Raw MRSI data is typically manually copied from the scanner's hard drive following an exam, processed offline, and rendered by an analyst, and the resulting screen captures are transmitted to PACS as DICOM Secondary Capture Image Storage SOP instances for radiologists to view in the reading room. Since the MRSI data are delivered to PACS separately from the rest of the exam, it may be necessary to notify the radiologist by e-mail, complicating their ability to read exams efficiently. Not only does this require extra workstations, storage, and personnel, but it results in inefficient delivery of results that are required for patient care. 

From a research perspective, the use of vendor-specific MRSI data formats hinders the development and validation of spectroscopic and metabolic imaging methods as there are limited software packages capable of reading, reconstructing, processing, displaying, and exporting data that are encoded in all of the most common data formats. This poses an obstacle to comparing data from multiple scanners and complicates the comparison of reconstruction, processing, and quantification algorithms using data from different scanner vendors. Though not widely implemented, the DICOM standard does define an information object definition (IOD) for encoding MRSI data [13], which could greatly simplify the use of MRSI if more widely adopted. Several freely available software projects address different aspects of these problems. jMRUI [16] is a closed-source package that supports reading, analysis, and visualization of MRSI data from multiple vendors as well as DICOM MRI and MRS data. The MIDAS package [17] is an open-source project that supports GE, Philips, and Siemens data and is distributed with an MRSI acquisition sequence implemented for each of these vendor platforms that MIDAS is capable of reconstructing and processing. TARQUIN [18] is an open-source package for spectral quantification that understands multiple vendor formats as well as the DICOM MR spectroscopy standard. Though these software packages provide needed functionality for the analysis of MRSI data, none of them provide a complete scanner-to-PACS workflow. The following sections describe the open-source software framework and application suite that were developed at UCSF to implement the DICOM MR spectroscopy (MRS) standard and to address MRSI analysis and workflow needs.

## 3. The SIVIC Software Suite

**Figure 2:**
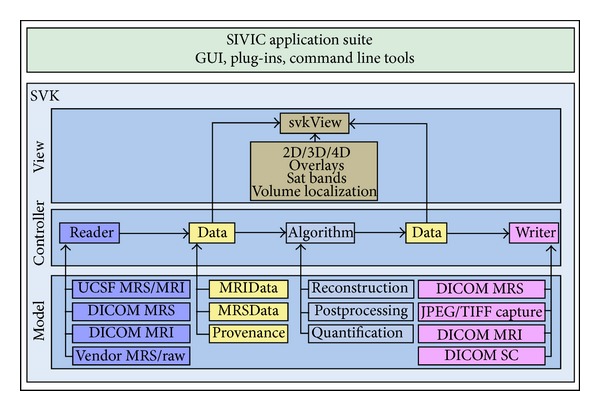
SIVIC software suite components. SIVIC applications (top) are built using the SIVIC Kit (svk) bottom. The svk is a C++ library representing a model, view, controller (MVC) design. View classes provide components that graphically display data and acquisition constructs represented by svkImageData objects (yellow). The controller layer utilizes svk IO (readers, writers) and svk algorithm classes to provide analysis functionality. The underlying svk model is represented by specific implementations of IO, algorithm, and data structure classes. Some specific examples of each class hierarchy are shown in the model (bottom box).

**Figure 3:**
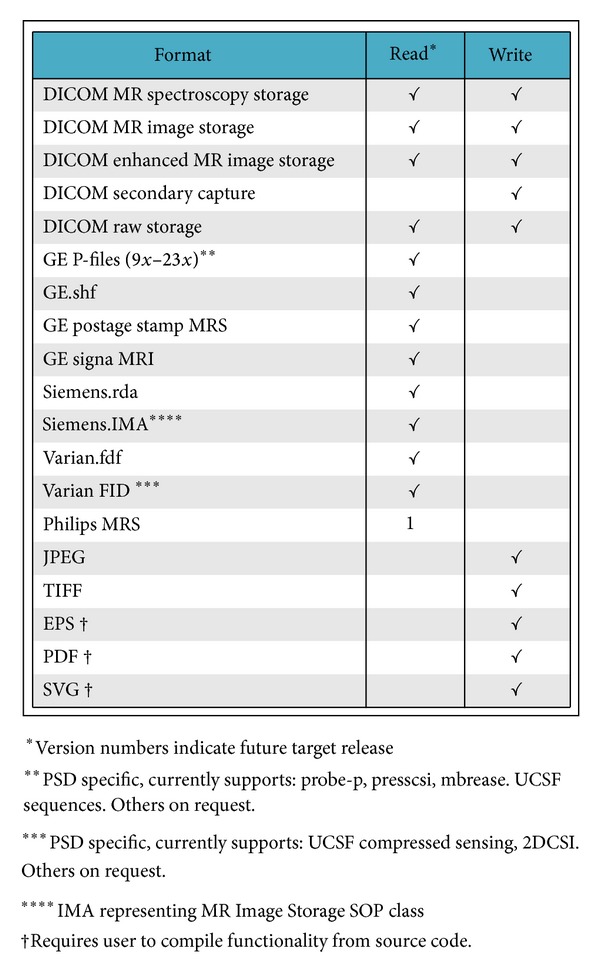
File formats supported by the svk IO layer are shown. SIVIC provides support for parsing raw data formats such as the GE P-file and Varian FID files, though interpreting the data may be sequence specific requiring customization to svk reader software classes. Version numbers indicate target SIVIC release to provide support.

SIVIC is an extensible, open-source, freely available, and cross-platform software suite designed to support all aspects of MRSI data analysis and visualization. It comprises a set of C++ libraries that support the various stages of analysis including data IO (input-output), algorithm pipelines, and visualization (Figure 2). This set of libraries is called the svk, for SIVIC Kit. Many of the svk C++ classes extend base classes from the visualization toolkit (VTK) for 3D visualization [19] or DCMTK [20], which provides low-level DICOM support. VTK is widely used in other medical imaging software enabling svk classes to be compatible with those packages. This compatibility is important for the development of SIVIC plug-ins to applications such as 3D Slicer [21]. The svk IO layer is a key component of SIVIC, enabling it to work with data from multiple formats and export data to the DICOM standard.  Figure 3 lists the data formats currently supported by SIVIC. The svk IO layer will be discussed in more detail below.

The classes in the svk libraries can be used to construct flexible MRSI applications that work with data from multiple vendor sources. In addition to providing these building blocks, the project provides a suite of applications that are built from the libraries. The most important application is the standalone SIVIC graphical user interface (GUI). This supports reading MRI and MRSI data, MRSI reconstruction, processing algorithms such as apodization, zero filling, and phasing, visualization of MRSI data and acquisition constructs such as the voxel grid, volume localization, and sat band placement and also supports exporting data to supported formats. The SIVIC GUI is also provided in the form of a plug-in for the OsiriX [15, 22, 23] open-source PACS and medical imaging package. This enables it to be used for visualization of MRSI data together with the storage management functionality provided by OsiriX PACS. A plugin for 3D Slicer [24, 25] is currently under development. SIVIC also provides command line tools [26] for converting between different file formats and for applying reconstruction, postprocessing, and quantification algorithms. Source-code and binary releases for OsX, Windows, and Linux are freely available from sourceforge: http://sourceforge.net/projects/sivic/. The software is released under a BSD license, which enables it to be freely used in open- or closed-source, free, or commercial applications. 

## 4. SIVIC Enabled DICOM MRSI Workflows

Current workflows for the delivery of quantitative MRSI data from the scanner to the reading room are inflexible and inefficient processes. Because standard reading workstations are incapable of rendering the high-dimensional MRSI data, they are typically rendered in the form of DICOM Secondary Capture Image Storage SOP instance reports and displayed as screen capture images. These images are limiting because they are static objects and cannot be further manipulated or analyzed. Even for product sequences that are reconstructed and analyzed on the scanner using vendor provided software, it is often desirable to create custom-tailored reports that focus on study-specific content and to generate reports from novel sequences or from analyses not supported by the vendor's native software. Providing customized DICOM secondary capture reports typically requires taking the data offline and using custom software algorithms. An added complication of such offline analysis is that non-DICOM MRSI data must be retrieved from the scanner using a separate workflow, for example, via SFTP [27], and must be stored separately from the DICOM exam. This results in a decoupling of the actual MRSI data from the rest of the exam and requires significant effort to maintain a searchable record for future retrieval. 

**Figure 4:**
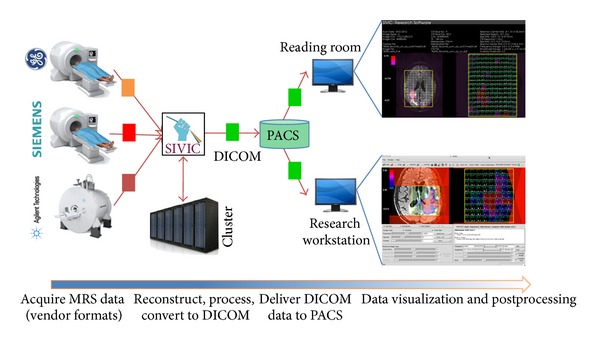
Generalized DICOM MRSI workflow. MRS data is acquired and encoded in vendor specific formats (red, orange, and pink). SIVIC tools reconstruct data and/or convert to DICOM format (green) to send to PACS. DICOM data can be retrieved for visualization in the reading room or on a research workstation for processing and visualization using the SIVIC GUI or command line tools.

In the following, on-scanner and off-scanner MRSI workflows that have been implemented at UCSF with SIVIC are described. A common enabling feature is the use of SIVIC to convert vendor-specific MRSI data to standard DICOM SOP classes that can be transferred from the scanner to PACS, managed with the rest of the exam data, and retrieved for review or additional analysis (Figure 4) using existing DICOM infrastructure or easily accessible open-source tools.

### 4.1. On-Scanner MRSI Workflow

**Figure 5:**
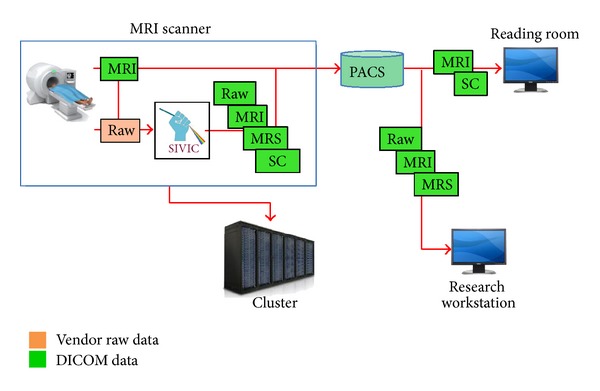
On-scanner MRSI workflow. SIVIC running on the scanner reads raw MRSI vendor data and anatomical DICOM MRI images. MRSI data is reconstructed and DICOM MRS, DICOM MRI metabolite maps, and DICOM secondary capture (SC) images are exported and sent to PACS. DICOM SC and DICOM MRI images are viewed in the reading room. DICOM MRI (anatomical and metabolite maps) and DICOM MRS images may be viewed on a research workstation running SIVIC or other DICOM applications. CPU intensive on-scanner reconstruction may require a computational cluster for real-time analysis during an exam.

**Figure 6:**
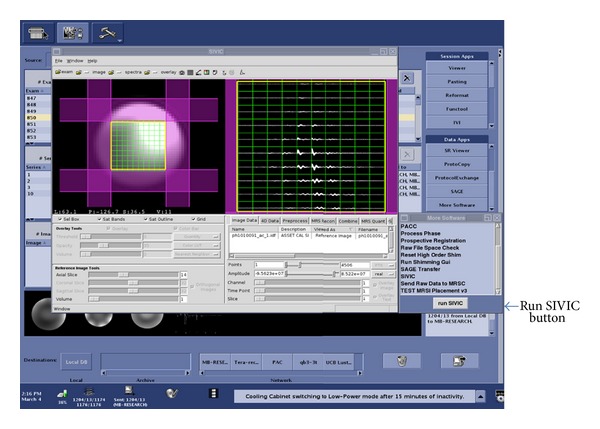
SIVIC GUI running on a GE 7T scanner console. Raw data from a phantom acquisition is shown in the right SIVIC panel. The left SIVIC panel shows the MRSI voxel grid spatially referenced to the reference image. The yellow box represents the PRESS volume localization, and purple regions represent sat bands. The SIVIC GUI is configured to run from configurable menu buttons on the scanner's operator console (right side).

**Figure 7:**
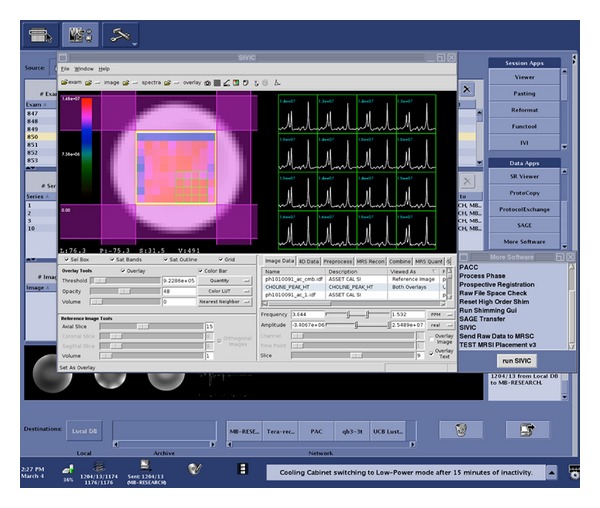
Phantom MRS data reconstructed and quantified using the SIVIC GUI on a 7T GE scanner console. The right panel shows spectra from the 16 selected voxels. The voxels are spatially referenced to the image in the left panel. The color overlay on the left is a metabolite map representing the choline peak height. The blue text above the spectra gives the exact value of the current overlay for each voxel.

This section describes a workflow for reconstructing and analyzing MRSI data directly on a scanner (Figure 5). Raw data are acquired and written to the scanner's file system in a vendor-specific file format. The SIVIC GUI is configured to start from customizable push buttons directly on the console. Once started, SIVIC loads the raw MRSI data and can optionally load 3D DICOM MR image storage reference images (Figure 6). Raw data from a phantom acquisition are shown in the right panel. The left panel shows the voxel grid spatially referenced to the reference image. The yellow box represents the PRESS volume localization, and sat bands are shown in purple. Once loaded into the GUI, the MRSI data may be preprocessed with apodization filters, zero-filled, reconstructed, and phased. The resulting spectra may then be quantified to obtain maps that represent the spatial distribution of various metabolites (Figure 7). For computationally demanding reconstructions, data are securely staged on a computational cluster [28] for batch processing using SIVIC's command line tools, and the results are returned to the scanner in near real time where they can be loaded for review in the SIVIC GUI. At this stage, the data are ready to be sent to PACS. If the data have been suitably prepared for radiological interpretation, a DICOM secondary capture report may be generated for review in the reading room. The quantified metabolite maps may be exported as DICOM MR Image Storage, or Enhanced MR Image Storage SOP instances, and the reconstructed MRSI data may be exported as a DICOM MR Spectroscopy SOP instance. The original raw data are encapsulated in DICOM Raw Data Storage SOP instances. 

**Figure 8:**
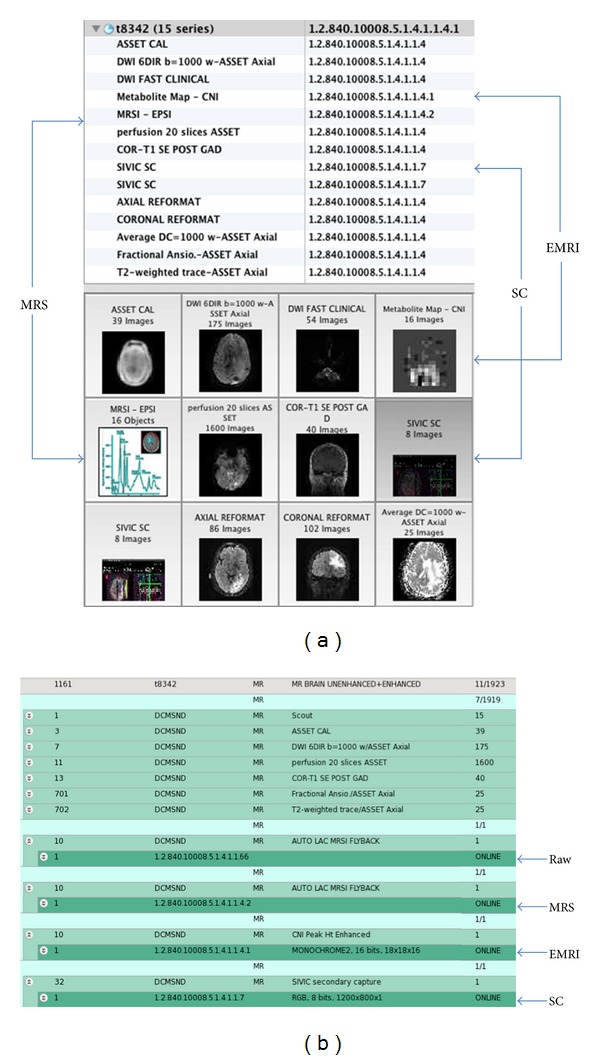
DICOM MRSI exam in OsiriX PACS (a) and DCM4CHEE PACS (b): Raw Data Storage SOP class (1.2.840.10008.5.1.4.1.1.66, RAW), reconstructed MRSI, MR Spectroscopy SOP class (1.2.840.10008.5.1.4.1.1.4.2, MRS), Secondary Capture SOP class (1.2.840.10008.5.1.4.1.1.7, SC), metabolite maps (Enhanced MR Image Storage SOP class (1.2.840.10008.5.1.4.1.1.4.1, EMRI).

**Figure 9:**
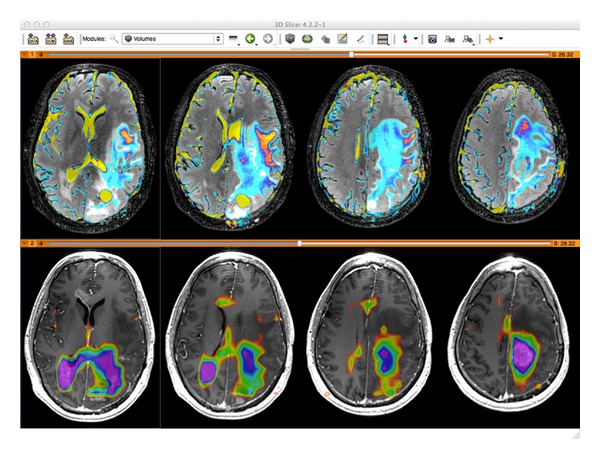
CNI metabolite maps (bottom color overlay) derived from MRSI data in SIVIC are exported as standard DICOM MR Image Storage SOP instances, which can be loaded into 3D DICOM image analysis software packages (shown here in 3D Slicer). Derived maps are thus amenable to multimodal analysis. The top panel shows ADC maps (color) on FLAIR images. The bottom panel shows the same anatomical locations on a T1 contrast enhanced image.

The complete exam, now in DICOM format, can then be transferred to an offline PACS system. Once in PACS, a radiologist may review the DICOM secondary capture report together with other anatomical or functional images. From a research workstation, the original raw or reconstructed data may be retrieved for additional processing and analysis.  Figure 8 shows an entire imaging exam including MRI, SC, MRSI, and raw data in OsiriX and DCM4CHEE [29] PACS. The entire exam including MRSI data and derived 3D metabolite maps is now archived in PACS, which is a major benefit for data management. A key point here is that the derived metabolite maps are 3D DICOM images which can be treated on an equal footing with other 3D imaging data to correlate MRSI with other data in a multimodal analysis (Figure 9). Specialized software such as SIVIC is still required for visualization of DICOM MRSI data, however, in principle any software package that implements the DICOM MR spectroscopy standard will be capable of interpreting it. The SIVIC plug-in for OsiriX permits the MRSI data to be visualized from within OsiriX PACS. Several other freely available software packages such as TARQUIN [18, 30] and jMRUI [16, 31] also support the reading of DICOM MRS data and provide capabilities that are complimentary to SIVIC. 

### 4.2. Off-Scanner MRSI Workflow

**Figure 10:**
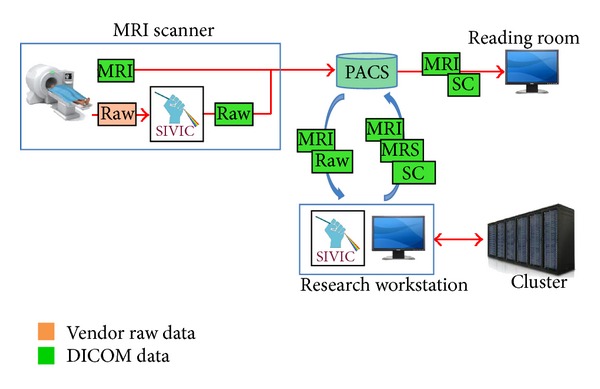
Off-scanner MRSI workflow. SIVIC command line tools running on the scanner convert vendor raw MRS data to DICOM Raw Data Storage SOP instances. Anatomical MRI and raw DICOM data is sent to PACS. A research workstation retrieves DICOM images from PACS. MRSI data is reconstructed and DICOM MRS, DICOM MRI metabolite maps and DICOM secondary capture (SC) images are exported and sent to PACS. DICOM SC and DICOM MRI data is viewed in the reading room. DICOM MRI (anatomical and metabolite maps) and DICOM MRS images may be viewed on a research workstation running SIVIC or other DICOM applications.

A workflow for reconstructing and analyzing MRSI data using an external workstation and transferring the resulting images is shown in Figure 10. In this scenario, raw data are encapsulated in Raw Data Storage SOP instances using the svk_create_dcmraw utility on the scanner and transferred to an offline PACS. The resulting DICOM raw data storage instances, together with the other DICOM data from the exam, are retrieved to a workstation where SIVIC tools process and reconstruct the data as described above. The DICOM SC report is sent back to PACS where it can be retrieved for review in the reading room. 

### 4.3. Workflow Discussion

MRSI data from patients with brain tumors are routinely acquired on GE MRI scanners at UCSF using product as well as novel acquisition methods developed in our research groups [32, 33]. These are converted to DICOM Raw Data Storage SOP instances using svk_create_dcmraw and pushed, together with other DICOM data, to a research DCM4CHEE PACS. 

**Figure 11:**
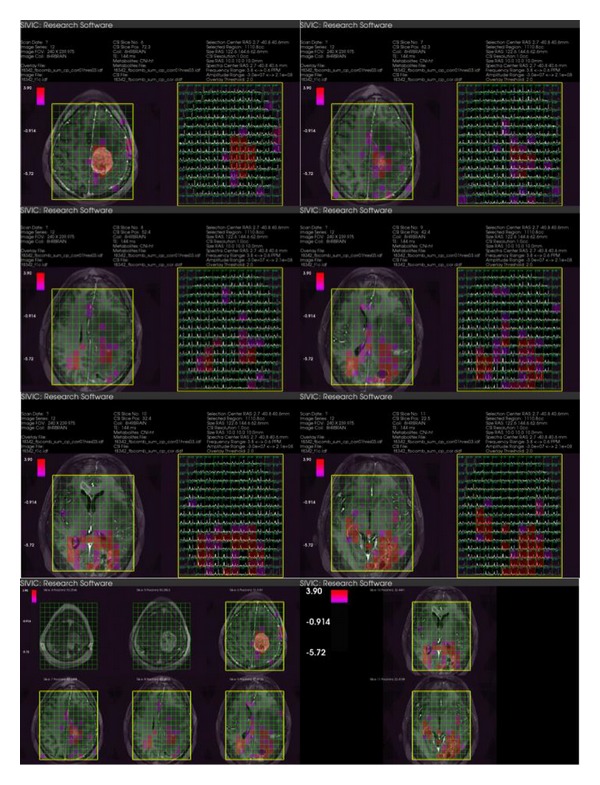
SIVIC generated DICOM secondary capture report for UCSF MRS exam. The series consists of 8 images shown here. The color overlay represents the choline to NAA index. Spatial referencing to T1 postcontrast image, volume localization (yellow), and sat bands (purple shading) are shown. The final two images are summary representations of the acquisition referenced to the anatomical images.

The DICOM exam is retrieved to a Linux workstation for processing. Details of the spectroscopic data processing pipeline are beyond the scope of this paper and are described here only at a high level. MRSI data is unencapsulated from the DICOM raw data storage object, and the file integrity is confirmed by the SHA1 digest. The unencapsulated raw data are converted to DICOM MR spectroscopy Storage instances with the command line svk_gepfile_reader utility. Apodization and zero filling as well as spatial and spectral Fourier transforms are performed within SIVIC. In addition to these methods, SIVIC supports zero and first-order phase correction, HSVD baseline removal, sum-of-squares coil combination, and peak height and integrated area metabolite quantification. Registration, segmentation, and other standard image processing algorithms are already implemented in other packages and are not reimplemented within SIVIC. Metabolite maps are exported from SIVIC as standard 3D images and can be processed using any number of available tools. The final processed MRSI data and MRI data are loaded into the SIVIC GUI in order to create a DICOM secondary capture report for radiological review as shown in Figure 11. The format of the report and its contents have been based on recommendations from neuroradiologists at UCSF who are involved in the treatment of patients with brain cancers. Over the past year approximately 400 brain MRSI reports have been sent to the UCSF clinical PACS for review using this method. 

A limitation of such workflows is that not all PACS implementations currently support the storage of Raw Data Storage SOP class or the MR Spectroscopy Storage SOP class, however many do, such as DCM4CHEE [34], OsiriX [35], Carestream [36], Philips [37], and Agfa [38]. Furthermore, reading workstations are still not capable of directly rendering MRSI data, which necessitates the use of DICOM secondary capture image reports. However, the ability to couple the raw and processed MRSI data with the DICOM record is a major benefit, making the data accessible to applications that implement the DICOM MRS standard. 

## 5. Modular Vendor Neutral MRSI Analysis Software

**Figure 12:**
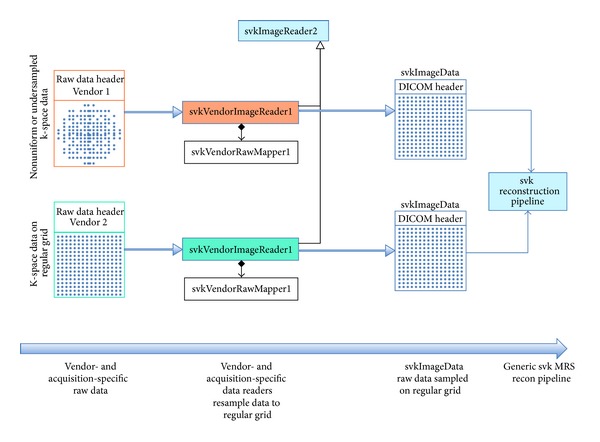
svk raw data readers handle acquisition-specific data reorganization. This includes vendor-specific header parsing and acquisition-specific data reordering and resampling. The output of an svkImageReader is always an svkImageData object, represented by a DICOM header and data sampled on a regular grid that is suitable for FFT-based reconstruction. This permits the use of a common set of independent downstream reconstruction and processing algorithms, independent of the acquisition sequence or vendor data format. An svkImageReaderFactory reads the raw data files to create the appropriate type of svkImageReader for the specific input format and acquisition type. Vendor- and acquisition-specific readers load data and associated mappers resample data to a regular grid.

Another major goal of SIVIC is to provide a flexible, vendor neutral MRSI analysis package that facilitates the validation of metabolic imaging methods and the dissemination of novel MRSI methods broadly within the community. The approach taken to achieve this is to separate vendor- and acquisition-specific details from generalized downstream reconstruction and analysis algorithms. All pipelines are thus divided into a data-reading component that is vendor and sequence specific, followed by a vendor and sequence neutral component representing the downstream processing pipeline as shown in Figure 12. 

Variability in data loading reflects differences in (i) data formats and (ii) acquisition methods. These differences are handled modularly within SIVIC's svkImageReader2 class hierarchy [39] in the following way. The process is split into two parts, reading the raw data file and interpreting its contents using a data mapping class. SIVIC implements readers for multiple vendor formats, and their responsibility is to parse a vendor's file format, but without making any interpretation of the content. Once the raw data has been parsed the data mapper is used to interpret the vendor- and acquisition-specific details such that the output of the svkImageReader2 (e.g., svkMrsImageData) consists of data sampled on a regularly spaced grid suitable for the Fourier transform reconstruction and acquisition-neutral processing [40]. Because the output of the readers has been standardized in this way, the SIVIC algorithms can be tested using data from multiple sources. 

The svk reader classes are modular at multiple levels. The vendor-specific readers only need to be implemented once per vendor data format. Mappers are more complex, yet the underlying algorithms utilized by the mappers to accomplish data reordering or resampling exist as separate svk algorithm classes that may be reused to accomplish the same task in similar data acquired on different vendor's scanners. For example, linear phase correction algorithms, required to correct for time delays in EPSI frequency sampling [32], may be used to make this correction on EPSI data from any vendor. This modularity is of great value and enables svk readers to be adapted for use with data from different vendors. At UCSF, this has enabled SIVIC software initially developed to read EPSI data acquired on a Varian animal scanner to be adapted easily for use on data acquired from a clinical GE scanner as studies transitioned from animal validation to human trials. 

## 6. Conclusions

The SIVIC software framework and application suite presented here comprise a widely accessible software package designed to facilitate the routine incorporation of MRSI data into imaging studies. This is accomplished by providing tools for converting MRSI data from nonstandard vendor-specific formats to the standard DICOM MR Spectroscopy SOP class. The use of this standard enables existing DICOM infrastructures to manage MRSI data together with other components of the exam, rather than requiring separate storage, transmission, and searching infrastructures. Two MRSI workflows that have been implemented at UCSF to analyze and deliver quantitative MRSI data from scanner to the clinical PACS and reading room in over 400 brain tumor exams were described. These workflows store GE raw data as DICOM Raw Data Storage SOP instances, reconstructed MRSI data as DICOM MR Spectroscopy Storage SOP instances, metabolic image maps as DICOM Enhanced MR Image Storage SOP instances and reports as DICOM Secondary Capture Image Storage SOP instances. DICOM MRSI data are maintained in a research PACS. This simplifies ongoing and retrospective analysis of imaging studies containing MRSI data. The encoding of derived 3D metabolite maps as DICOM MR Image Storage SOP instances enables them to be used by standard DICOM image analysis software, thus providing a straightforward mechanism to integrate metabolic data with other anatomical and functional imaging data as part of a multimodal analysis. 

The use of the DICOM MR Spectroscopy SOP class to encode MRSI data increases data accessibility to any application that implements the DICOM MRS standard. As has been demonstrated here, this allows MRSI data to be managed by conventional PACS solutions and enables MRSI analysis software to be used for evaluation of data from multiple sources. SIVIC extends the interoperability to data originally encoded in vendor-specific formats and thus enables a common set of software algorithms and visualization tools to be used with data from multiple sources. Tools developed on one scanner platform can thus be relatively easily ported to other scanner platforms. This facilitates the transition of methods from animal models to human models and streamlines the use of MRSI analysis in multicenter trials. Other dynamic imaging modalities can also benefit from the type of high dimensional visualization tools used here for display of MRSI data. For example, MR perfusion studies track the time evolution of contrast in a 3D volume, and SIVIC has been adapted to display such data sets both as 3D arrays of time curves as well as 3D maps representing derived perfusion parameters.

The distribution of SIVIC as a free open-source software package that runs on all major operating system has been shown to foster interinstitutional MRSI research studies as research MRSI acquisition sequences can be distributed together with the software required for reconstruction and visualization of data acquired with novel MRSI methods. These collaborations provide important feedback for the project that acts to stabilize the distributions and improve functionality. The project encourages community participation and welcomes collaborative input.
